# Ein Lob der Habilitation

**DOI:** 10.1007/s00048-024-00393-2

**Published:** 2024-07-16

**Authors:** Reinhard Kahle

**Affiliations:** 1 Carl Friedrich von Weizsäcker-Zentrum, Universität Tübingen, Doblerstr. 33, 72074 Tübingen, Deutschland; 2 DM und Center for Mathematics and Applications (NOVA Math), NOVA School of Science and Technology (NOVA FCT), Universidade Nova de Lisboa, Lissabon, Portugal

Wenn man den deutschen Universitäten das Urteil ausstellt, im Hinblick auf die Situation des Mittelbaus in einer Krise zu stecken, dürfte man sich zurecht den Vorwurf einer Untertreibung einhandeln. Das dtv-Lexikon von 2006 schreibt unter dem Stichwort Mittelbau: „eine Gruppe akadem. Lehrer an den Hochschulen der BR Deutschland zwischen den habilitierten Hochschullehrern u. den Assistenten, bestehend aus Akademischen Räten, Studienräten im Hochschuldienst, Studienprofessoren u. a. Voraussetzung ist Promotion, nicht die Habilitation.“ (Anonym 2006). Heute dürften wir einen Großteil der zum Mittelbau gehörenden Angestellten einer Universität unter den mit einem unscheinbaren „u. a.“ genannten „akadem. Lehrern“ finden – nämlich diejenigen, die statt einer Ratsstelle nur eine befristete Stelle haben. Und wenn in diesem Eintrag auf die Habilitation als Voraussetzung für Professuren verwiesen wird, stellt sich die Frage, ob diese Habilitation heute nicht bereits ein Relikt eines überkommenen Universitätsverständnisses ist. Tatsächlich stellt die Habilitation selbst einen Teil des Problems dar: Sie kann als ein Befristungsgrund herhalten, der im Mittelbau Angestellte in prekären Anstellungsverhältnisse gefangen hält. Es gibt noch eine Reihe anderer Gründe, die einer Habilitation den Anstrich einer veralteten, geradezu nur zur Abschreckung dienenden Hürde in der Universitätshierarchie geben. In diesem Diskussionsbeitrag wollen wir zuerst kurz einige Probleme der Habilitation skizzieren. Anschließend wollen wir aber eine Lanze für diese Qualifikationsform brechen, besonders im Hinblick auf die Juniorprofessur als aktuell propagierte Alternative. Dabei soll auch deutlich werden, dass die Habilitation gerade in Fächern wie der Wissenschaftsgeschichte eine besondere Rolle spielt, weil sie unter Umständen das letzte Mittel ist, das „Überleben“ solcher kleinen Fächern zu sichern.

Die Habilitation als formale Qualifikation für die Berufung auf eine Professur ist eine vergleichsweise junge Institution in der (deutschen) Universitätsgeschichte. Sie wurde Anfang des 19. Jahrhundert mit der Gründung der Berliner Universität eingeführt und von „nacheifernden Universitäten“ übernommen (Boockmann 1999: 193; Koch 2008: 126f.). Das heißt aber weder, dass es vorher keine vergleichbaren Qualifikationshürden gegeben hätte, noch, dass dies ein rein deutsches Verfahren wäre. Schon Kants Karriere begann in einer Weise, die sich in der Sache wohl kaum vom Werdegang eines habilitierten Privatdozenten unterscheidet (siehe die Beschreibung in Koch 2008: 128f.). Traditionell war der Doktortitel der Ausweis für die Lehrbefähigung. Mit dem Niedergang des Niveaus der Promotion ergab sich aber der Bedarf einer neuen Qualifikationsprüfung (Rasche 2013; Koch 2008: 180f.).

Auch andere Länder kennen ähnliche Institutionen, wenn auch häufig mit deutlichen Unterschieden zur deutschen Habilitation und eventuell sehr viel jüngeren Datums. So kennt Frankreich seit 1984 die *Habilitation à diriger des recherches* (HDR), die nicht von der eigenen Universität begutachtet wird (Fröhlich 2005). Portugal kennt eine *Agregação*, die auf der Ebene der außerordentlichen Professur (*professor associado*) nötig ist, um sich auf einen Lehrstuhl (*professor catedrático*) bewerben zu können.

Die deutsche Habilitation hat aber zwei Spezifika, die wir besonders hervorheben wollen: Sie wird vor einer Fakultät – üblicherweise „der eigenen“ – verteidigt, und sie kommt in aller Regel mit der Lehrbefugnis für ein Fach, in dem man anschließend als Privatdozent oder Privatdozentin an der prüfenden Fakultät eigenverantwortlich, aber ohne unmittelbaren Vergütungsanspruch lehren darf.

Die Abhängigkeit von (der Gunst) der Fakultät ist sicher einer der Hauptkritikpunkte, denen sich die Habilitation ausgesetzt sieht. Man ist faktisch verpflichtet, es in den Jahren vor der Prüfung allen Angehörigen der Fakultät, insbesondere auf der Ebene der Professuren, recht zu machen, da diese andernfalls ohne große Rechenschaftspflicht bei der Prüfung gegen die Habilitation stimmen könnten. Hat man es sich – auf welche Art und Weise auch immer – mit einem „Platzhirsch“ an der Fakultät „verscherzt“, kann dieser die weitere Karriere praktisch zunichtemachen. Da in einem Habilitationsverfahren von Fakultäten sehr häufig „mitgeprüft“ wird, wie man sich in diese Fakultät eingebracht hat, sind Habilitationen an einer anderen Universität die absolute Ausnahme.

Zuweilen findet man sich an den Hauptmann von Köpenick erinnert, wenn formale Regularien für die Zulassung zur Habilitation Lehrerfahrung voraussetzen, gleichzeitig an einer Fakultät aber nur mit der Habilitation gelehrt werden darf. Auch wenn solche Regeln pragmatisch unterlaufen werden, sieht man sich bei einer Habilitation nicht ganz zu Unrecht einer Prüfung gegenüber, die zum Teil willkürliche Bewertungskriterien beinhaltet.

Solange die Habilitation formale Voraussetzung für die Berufung auf eine Professur war, kam noch ein anderes Problem mit hinzu. Strenggenommen schloss diese Bedingung Bewerbungen aus dem Ausland aus. Dem traten die Universitäten dadurch entgegen, dass ein Engagement an ausländischen Universitäten in der Regel bereits als „habilitationsäquivalente Leistung“ gewertet wurde (man beachte aber, dass z. B. Frankreich und Portugal heute in der Regel noch immer darauf bestehen, dass die nationale Habiliationsqualifikation vor einer Bewerbung auf eine entsprechende Professur erbracht werden muss). Wer also bereit war und die Gelegenheit bekam, von Deutschland aus für eine Weile ins Ausland zu gehen, konnte sich die „ungeliebte“ Habilitation ersparen.

Obwohl die Habilitation in früherer Zeit durchaus „in jungen Jahren“ erfolgen konnte, haben die allgemeine Entwicklung hin zu Studienzeitverlängerungen, wieder gestiegenen Anforderungen an die Promotion, vor allem aber auch Erwartungen an eine „zeitlich gereifte“ Habilitation dazu geführt, dass das Eintrittsalter in die Professur in Deutschland mit der Zeit immer weiter stieg. Wir nehmen an, dass dies der Hauptgrund war, warum „von oben“ – das heißt konkret zum Beispiel von der Deutschen Forschungsgemeinschaft – Anstrengungen unternommen wurden, die Habilitation – durchaus gegen den erklärten Willen der Fakultäten – „abzuschaffen“.

Aber was sollten die Alternativen sein? Als erstes gilt es zuzugestehen, dass es sinnvoll ist, der Berufung auf eine Professur eine Qualifikationsprüfung vorausgehen zu lassen, so ärgerlich das auch sein mag. Eine Doktorarbeit, auch wenn ihr Niveau gegenüber dem 19. Jahrhundert inzwischen wieder deutlich angehoben wurde, reicht deshalb nicht aus, weil bei ihrer Prüfung Lehrkompetenzen überhaupt keine Rolle spielen. Als Ausweg wurde – in Anlehnung an amerikanische *tenure track*-Professuren – die *Juniorprofessur* „erfunden“. Diese wird – wie andere Professuren auch – von der Fakultät ausgeschrieben, und es findet ein normales Berufungsverfahren statt. Man hat dann eine gewisse Zeit (zum Beispiel fünf Jahre), um sich unter anderem in der Lehre zu bewähren und *kann* dann dauerhaft eine Professur erhalten. Macht das die Habilitation obsolet? Manche wünschen sich das – oder sehen es sogar schon realisiert, „weil wir ja nicht mehr klassisch habilitieren“ (Stark-Watzinger 2023). Doch die Sache mit den Juniorprofessuren als Alternative hat einen substanziellen Haken: Diese Professuren müssen von einer Fakultät ausgeschrieben werden! Nun können Fakultäten nicht dazu gezwungen werden, Juniorprofessuren einzurichten (obwohl es hier zuweilen durchaus unsanften Druck von Rektoraten und „von außen“ geben mag) – noch weniger dazu, die regulären Professuren über den „Umweg“ einer Juniorprofessur zu besetzen. Fachbereiche haben das Recht, bei der Besetzung ihrer Professuren weiterhin die Habilitation als Qualifikationsmerkmal in die Bewertung einfließen zu lassen. Das ist wohl tatsächlich mehr die Regel als die Ausnahme, gerade in „konservativen Fächern“ wie zum Beispiel Jura. Als Konsequenz wird die Juniorprofessur als „Standardqualifikation“ für eine volle Professur nur halbherzig umgesetzt (siehe dazu die allerdings schon etwas zurückliegende ausführliche Diskussion in Dehrmann & Hausmann 2018). Vielleicht ist dieser Weg ja auch gar nicht der richtige.

Das Hauptproblem ist, dass es an den Fakultäten der Universitäten liegt, eine Juniorprofessur in einem bestimmten Fach auszuschreiben oder nicht. Zwar kann man sich jetzt „überall“ bewerben und wäre nicht mehr wie bei der Habilitation mehr oder weniger fest an seine eigene Fakultät gebunden. Wenn aber in einem spezifischen Fach keine Juniorprofessur ausgeschrieben wird, läuft nicht nur die Karriere *einer* Person dieses Fachs ins Leere, sondern die Karriere *jeder* Person, die in diesem Gebiet noch nicht etabliert ist. In letzter Instanz drohen damit ganze Fächer „ausgetrocknet“ zu werden – allein, weil keine Fakultät dazu Stellen auszuschreiben gewillt ist.

Hier wird ein (erster) Grund sichtbar, warum die Habilitation auch heute noch ihre Existenzberechtigung hat. Als Qualifikationsprüfung, die vom Kandidaten beziehungsweise von der Kandidatin selbst beantragt werden kann, unterliegt sie thematisch nicht einer vorhergehenden Entscheidung der Fakultät. Damit kann durch eine Habilitation ein Fach aus Eigeninitiative „am Leben erhalten werden“. Da die Habilitation mit der Lehrbefugnis einhergehen sollte, sollte dieses Fach auch weiterhin in der Lehre vertreten sein. Natürlich bleibt die finanzielle Situation prekär, da Privatdozenturen unbezahlt sind. Doch wenn man auf eine Juniorprofessur angewiesen wäre, die schlicht nicht ausgeschrieben wird, wäre man gezwungen, die Universität zu verlassen – und damit droht unter Umständen sogar das Fach akademisch zu verschwinden. Tatsächlich betrifft die Wahlfreiheit nicht nur das Fach, sondern auch das spezifische Forschungsthema, das in einer Habilitation gewählt wird. Zu Ende gedacht stehen sich bei der Alternative Habilitation oder Juniorprofessur Konzeptionen gegenüber, bei der in der Habilitation selbstbestimmte Forschung mit einer von Hochschulleitungen zu verantwortenden, dirigistischen Kanalisierung von Ausschreibungsgebieten einer Juniorprofessur konkurriert.

Es gibt noch einen zweiten Grund, der für den Erhalt der Habilitation spricht: Die Besetzung einer Juniorprofessur erfolgt nach einer Ausschreibung, bei der es naturgemäß mehrere Bewerbungen geben kann. Das anschließende Auswahlverfahren ist ein *konkurrierendes*; das heißt die Bewerbungen werden miteinander verglichen und die „beste“ sollte sich – zumindest der Theorie nach – durchsetzen. In der Habilitationsprüfung wird man dagegen – wieder der Theorie nach – nach *absoluten* Maßstäben geprüft, ohne dass man sich eines Vergleichs mit anderen Bewerbungen ausgesetzt sieht. Soweit die Juniorprofessur die Eignung für die Besetzung einer (vollen) Professur prüfen soll, ist nicht einzusehen, warum diese nur Personen offenstehen soll, die sich in einem konkurrierenden Wettbewerb durchgesetzt haben – über eine „Qualifikation“ sollte man unabhängig von möglichen Konkurrenten verfügen können. Genau das wird durch die Habilitation als Qualifikationsnachweis ermöglicht.

Wenn man sich in einem Gebiet mit einem „übersättigten Markt“ von qualifizierten Kandidaten und Kandidatinnen wiederfindet, ist es durchaus keine Schande, der besseren Konkurrenz bei der Bewerbung um eine Juniorprofessur zu unterliegen. Doch soll man deshalb keine Möglichkeit haben, seine Qualifikation in dem Gebiet unter Beweis stellen zu dürfen? Dem Argument, dass man aufgrund der besseren Konkurrenz ja ohnehin keine Chance auf eine Professur habe und deshalb „zu Recht“ aus dem Markt gedrängt würde, mögen manche Leute vielleicht etwas abgewinnen wollen („Wissenschaftler müssen frühzeitiger wissen, ob sie eine dauerhafte Perspektive in der Wissenschaft haben“; Stark-Watzinger in Stratmann 2023). Wenn aber „der Markt“ nur dadurch beschränkt ist, dass sich Universitäten nicht genötigt fühlen, Stellen in spezifischen Fächern auszuschreiben, bleiben auch die qualifiziertesten Personen auf der Strecke.

Das letztgenannte Szenarium kann nun ganz besonders kleine Fächer – wie es auch die Wissenschaftsgeschichte ist – treffen. Wenn man nicht will, dass das Überleben dieser Fächer vermeintlich strategischen Überlegungen der lokalen Fakultäten, der Universitätsrektorate oder gar sogenannter Exzellenzstrategien überlassen bleibt, hat man mit der Habilitation ein Mittel, um diese Fächer am Leben zu erhalten.
